# Prevalence of Bovine Trypanosomosis and Its Associated Risk Factor in Hawa Galan District, Kelem Wollega Zone of Ethiopia

**DOI:** 10.1155/2021/4531586

**Published:** 2021-11-30

**Authors:** Girma Tsegaye, Belay Abebe, Geremew Haile

**Affiliations:** ^1^Jimma University, College of Agriculture and Veterinary Medicine, P.O. Box: 307, Jimma, Ethiopia; ^2^Wollega University School of Veterinary Medicine, P.O. Box: 395, Nekemte, Ethiopia

## Abstract

An analytical cross-sectional study was performed between November 2015 and April 2016 at Hawa Galan woreda, Kelem Wollega Zone, Oromia Region, Ethiopia, to estimate the proportion of cattle with trypanosomosis and to evaluate the elements associated with the prevalence of bovine trypanosomosis. A haemoparasitological examination of the Buffy coat method was implemented to determine the proportion of trypanosomosis-positive cattle. A study population of 428 bovines was randomly selected from three peasant associations (PAs) and tested for the presence of the disease. Findings of the laboratory results indicate that among all animals tested at the study time, 26 (6%) animals were positive for the disease. Among the positive results, the proportion of trypanosome species was *Trypanosoma congolense* 18 (69.3%) and *Trypanosoma vivax* 8 (30.7%). The prevalence at the selected PAs was 12.5%, 3.8%, and 3% for Lemlem, Madawalkituma, and Ifajiru, respectively. From this finding, the relationship has a statistically significant variation (*P* < 0.05) among peasant associations and body condition state, and the proportion was significantly greater (*P* < 0.05) in animals categorized under poor body condition score. The relationships between age and sex of cattle show no statistically significant variation among them (*P* > 0.05). This study indicates that the proportion of trypanosomosis was greater in anemic (PCV<24%) cattle than nonanemic (PCV≥24%). Therefore, based on this finding, the proportion of bovine trypanosomosis is evident that can cause a major effect on the health of cattle in the study population in the study area. So, effective control methods could be applied to decrease the disease and its related economic loss.

## 1. Introduction

Trypanosomosis is a vector-transmitted infection of animals triggered by blood borne parasites called as trypanosomes. Trypanosomosis is found in places where its vector tsetse fly is present in Africa, from latitude 15°N and 29°S. The most common species of trypanosomes causing disease in cattle are *Trypanosoma congolense*, *Trypanosoma vivax*, and *Trypanosoma brucei*. Trypanosomes, predominantly, *T. vivax*, can also be transmitted mechanically via biting flies, and as a result, the organism is present in some parts of Central and South America where there are no any of its vectors [1]. In Africa, the vectors occupy extensive variety of environments extending beyond 10 million Km^2^ that represents about 37% of the African continent and harming the economies of 38 countries including Ethiopia [2].

AAT induces major economic losses in cattle production due to anemia, loss of power, and poor body condition. If disease remains untreated, it is lethal [3]. It is among the most top-ranked infections of animals that hinder the farming system in underdeveloped sub-Saharan African countries as well as in our country Ethiopia [4]. The outcome of a disease is manifested by a reduction in productivity gains, reduced rates of calving, and a greater risk of death, as reported according to the FAO [5].

Trypanosomosis is among one of the foremost obstacles to animal growth and agronomic gain in Ethiopia posing adverse effect for the whole economy and for nourishment independence exertions of the nation in specific. Vector-transmitted trypanosomosis is limiting the use of fertile land that is suitable for agricultural production located in the west and southwest of Ethiopia. This represents an area of about 180,000–200,000 km^2^; 14 million head of cattle, 7 million equines, 1.8 million camels, and also, some proportion of small ruminant animals are at risk of contracting the disease [6].

The major trypanosome types causing the disease in animals in the Ethiopia are *T. congolense, T. vivax*, and *T. brucei*, in bovines and small ruminants (shoat), *T. evansi* in camel, and *T. equiperdum* in horse [7]. The proportion of trypanosomosis will be influenced by the degree of contact, presence of diseased cattle, insect reservoir, and seasons [8]. Various methods are available for diagnosis of the disease such as wet mount, buffy coat examination, and the polymerase chain reaction (PCR) technique. The PCR test shows better sensitivity than classical parasitological approaches. Applications of PCR result in a correct amplification of products from the particular disease agent of interest, which can inform treatment options to be performed as soon as is practical in the field that facilitates improved control programmes [9].

Different preventive methods have been implemented to control trypanosomosis in cattle in place where the disease presents. Nowadays, the best method of prevention for trypanosomosis is mostly based up on the control of the disease vector that includes spraying of insecticides against the tsetse vectors and deploying of tsetse-fly traps [10]. Vaccine development for the disease is impossible due to the capability of the causative agent to escape animals' defense mechanism through the different way that have a joining effect by antigenic polymorphism and immune suppression [11].

Another effective method of disease prevention is to develop and achieve trypanotolerance cattle breeds to maintain productivity in place of great fly burden. The introduction and keeping of trypanotolerant West African taurine cattle breeds seem to be an alternative biological method for preventing clinical trypanosomosis and, thus, reducing economic losses for the animal holders. Trypanotolerance is a feature of both West African long-horn and short-horn *Bos taurus* breeds such as the N'Dama and Baoule breeds [12]. The principal drugs currently available to manage AAT in trypanosome endemic areas are isometamidium chloride, diminazene aceturate, and homidium; however, the development of drug resistance to the disease becomes a major problem toward control of the disease [13–15]. Bovine trypanosomosis has been investigated widely in different parts of Ethiopia [16–20]. However, there is a scarcity of information on the prevalence of bovine trypanosomosis and its risk factors in Hawa Gelan district, Kelem Wollega Zone of Ethiopia. Therefore, this research aims to estimate the proportion of trypanosomosis-positive cattle which are located in the Hawa Galan district of Ethiopia and to determine the influence of selected risk factors to cattle testing positive.

## 2. Materials and Methods

### 2.1. Study Area

The research was performed in Hawa Galan woreda that is located in Kelem Wollega Zone, in the western part of Ethiopia. It is approximately 630 km from Addis Ababa, the capital city of Ethiopia, and about 27 km from Dembidolo town. Geographically, Hawa Galan woreda is located 08°34′N and 35°59′E, with elevations of 1200 to 2200 meters above sea level. The yearly mean temperature ranges from 21°C to 24°C, and the annual rainfall range is 500–900 mm. The livestock populations of the woreda are estimated to be 60,202 cattle, 30,120 sheep, 35,120 goats, 38,386 poultry, 4,693 horses, and 5,232 donkeys, and the livestock are free-grazing. The agricultural scheme of the place is mixed farming where 87% of the entire inhabitants are involved in agriculture [21].

### 2.2. Study Population

The research was conducted on 428 randomly selected cattle, from three kebeles. Study animals were managed under an extensive system in which cattle are placed within a free-grazing system. Among the selected cattle, 120 were from Lemlem, 100 from Madawalkituma, and 208 from Ifajiru. Inspection and assessment of body condition were accomplished during sample collection. The age of each animal was classified into three age categories: less than 2 years of age, between 2 and 5 years, and greater than 5 years of age. Depending upon the body condition score, they were classified into poor-, medium-, and good-scored animals [22].

### 2.3. Sample Number Estimation

For simple random sampling, the sample size determination, and sampling approach, the number of animals required for the study was determined by the following formula [23]:

(1) $$\begin{array}{c} n=\frac{1.96^{2}}{d^{2}}\times P\text{exp}\left(1-\text{Pexp}\right), \end{array}$$

where *n* = required sample size, Pexp = expected proportion, and *d* = desired absolute precision (0.05). Size of the sample is assessed using 95% level of confidence, 12.4% expected proportion, and 0.05 required absolute precision. Thus, 167 cattle are sampled. Nevertheless, to increase the precision, the sample was increased to 428.

### 2.4. Study Design and Sampling Techniques

Analytical study known as a cross-sectional study was performed from November 2015 to April 2016 in order to estimate the proportion of trypanosomosis and to assess linked risk factors in the study animals. The animals were selected randomly and restrained by farmers for sampling.

### 2.5. Study Methods

A blood sample was collected by puncture of the ear vein using a lancet, and the blood sample was collected in heparinized microhaematocrit tubes. The buffy coat technique was used for diagnosis to identify trypanosome species based on their movement in wet film preparations as previously described [24]. The detailed buffy coat procedures were as follows.

The tube was filled to at least ¾^th^ of its volume and was sealed at one end with crystal seal [25]. One end of the capillary tube is sealed and centrifuged at 12,000 rpm for 5 minutes to isolate the blood cells and to obtain trypanosomes using centrifugal powers. After that, the packed cell volume of red blood cells is calculated by using a haematocrit reader. The length of the packed red blood cell column is explained as a percentage of the whole volume of blood. Animals that have PCV less than 24% were categorized as anemic [1]. The capillary tubes were broken approximately 1 mm below buffy coat and expressed on a microscopic slide, mixed, and covered with a 22 × 22 mm cover slip. The preparation was observed under a 40x objective of microscope by the dark ground buffy coat method to examine the presence of the trypanosomes based on their motility [26].

### 2.6. Data Management and Analysis

To undertake statistical analysis, all of the data were entered into Microsoft Excel spread sheet and then analyzed by using SPSS version 20.0. The number of infected individuals was divided by the number of animals sampled and multiplied by 100 to calculate the prevalence of bovine trypanosomosis. Statistically significant relationships between variables were said to present with calculated *P* < 0.05 and 95% confidence level, while the effect of the different risk factors associated with trypanosomosis was observed using multivariate logistic regression.

## 3. Results

Among the total of 428 animals examined from three peasant associations, 26 animals were identified as positive for the disease with an overall proportion of 6% with 0.04 and 0.08 lower and upper 95% CI, respectively (Table 1).

Of the cattle that tested positive for trypanosomosis, 3 (1.8%), 7 (5.38%), and 16 (11.9%) were observed to have good, medium, and poor body condition status, respectively, and there were statistically significant variations (*P* < 0.05) among body condition scores (Table 2).

Among 428 cattle examined, 209 were male and 219 female, of which 14 (6.7%) and 12 (5.48) were found to be positive for trypanosomosis, respectively; however, there was no statistically significant difference (*P* = 0.598) value among sex classes as depicted in Table 3.

Data analysis among different age classes of cattle showed no statically significant difference (*P* > 0.05) as depicted in Table 4.

In the study population, 220 (51.4%) and 208 (48.6%) were anemic (PCV < 24) and nonanemic (PCV ≥ 24), respectively. Among anemic cattle, 23/220 (10.5%) and 197/220 (89.54%) were found to be parasitemic and aparasitemic, respectively (Table 5).

Out of 428 cattle examined, 18 (69.3%) animals were positive for *T. congolense* and 8 (30.7%) cattle were positive for *T. vivax.*

A logistic regression was employed to ascertain the effects of age, sex, and body condition score on the likelihood to evaluate their association to the disease. Accordingly, the body condition score was found to be a significant effect on the proportion of cattle trypanosomosis with less proportion in cattle with good body condition compared to the other one (OR = 0.14, 95% CI = 0.04–0.5, *P* = 0.003), while males were 1.34 times more likely to exhibit the disease than females (Table 6).

## 4. Discussion

Dissemination of circulating species of trypanosomes that infect cattle in Ethiopia differs significantly from area to area. The current finding indicated the whole proportion of 6% (26/428) at the Hawa Gelan District, Kelem Wollega Zone of Ethiopia. The report is in agreement with previous studies which reported an overall proportion of 6.86%, 6.90%, and 7.81% in LaloKile district of Kelem Wollega Zone, in Chena district of South West Ethiopia and in Guto Gida woreda of East Wollega Zone, respectively [27–29]. This relationship in proportion with the present finding could be due to the high distribution of this parasite and its vector throughout the country and decreased control method. Besides this, the finding is low when related with former findings, 16.9% in Sayo Woreda of Kelem Wollega Zone [30] and 27.5% in the district of Arba Minch Abraham and Tesfaheywet [31]. The comparatively lower proportion of trypanosomosis in the current study could be because of the variations in agro climate, which is less favorable to the vectors growth and multiplication and low fly-animal interactions. Moreover, there were the trypanosome and tsetse control programs experienced in the western parts of Ethiopia including Hawa Gelan district which might predispose to the low proportion.

During the study period, the proportion of the disease was evaluated with respect to sex, 12 (5.48%) and 14 (6.7%) of females and males testing positive, respectively. Slightly greater infection proportions were detected in male than females in the current study; however, the variation was not statistically significant. This finding is in line with a previous study that reported slightly greater (8.17%) infection proportion in female than male and 6.02% which was not significantly different between the both sexes [32].

Associations of trypanosomes with selected peasant associations were also assessed. The proportion of cattle's trypanosomosis among the kebeles was statistically significant (*P* < 0.05) even though they are in the same agroecology. Higher proportion was recorded in Lemlem (12.5%) followed by Ifajiru (3.8%) and Madawalkituma (3%). The highest proportion in Lemlem PAs might be due to the accessibility of these PAs to the Mojo River, which harbor the diseases vector (tsetse fly) compared to other PAs.

From these findings, *T*. *congolense* and *T*. *vivax* were found as the two predominant trypanosome species; accordingly, their finding in the Hawa Galan District was 69.30% (18/26) and 30.70% (8/26), *T. congolense* and *T. vivax*, respectively. This result agreed with the finding from the East Wollega Zone *T*. *congolense* (72.73%) and *T. vivax* (27.27%) by Tafese et al. [33]. This higher ratio of *T. congolense* could be due to the high presence of the major cyclical vector, while *T. vivax* is additionally spread mechanically by biting flies than by vectors. In addition, the high proportion of *T. congolense* infection in cattle may be because of the great number of serodems of *T. congolense* as related *to T. vivax* and production of immune reaction to *T. vivax* by the diseased animals [25]. Based on finding of Abebe [7], *T. congolense* and *T. vivax* are the major predominant trypanosomes that infected cattle in the tsetse habitat and tsetse-free place of our country.

Eventhough the difference in proportion of disease among age categories was not significant (*P* > 0.05), the present findings revealed a high proportion in those cattle aged >5 years (7.9%) followed by animals between 2 and 5 years of age (5.9%) and less than 2 years of age (2.4%). This could be due to the fact that aged cattle at the place are typically kept in the forest for an extended duration for feeding, which might make them prone to vector contact compared to young cattle [34]. Based on finding to Torr *et al.* [35], this could also be because of the reason that the vectors of the disease were more attracted to the odor of older cattle and less attracted to younger animals.

In this study, the proportion of disease according to body condition of cattle was evaluated, and the proportion was greater in cattle with low body condition (11.9%) followed by medium (5.38%) and good (1.8%) body status with a statistically significant variation (*P* < 0.05). This is in line with the work of Teka et al. [36] performed in selected areas of Arbaminch, Ethiopia, presenting that the chief proportion follows the same results obtained here (body condition: poor- 12.22%; medium- 2.32%, and good- 2%). This could be associated to the prolonged nature of the infection. According to Radostits et al. [37], the highest proportion in poor-body-conditioned animals was due to the debilitating nature of the disease. The finding of the proportion of disease was higher in anemic (PCV<24%) cattle than nonanemic (PCV ≥ 24%). This might show that the disease cause a decrease of PCV.

The average PCV result of diseased and nondiseased cattle was also evaluated, and trypanosome disease and average PCV acquired among them had a statistically significant variation (*P* < 0.05), and it was lower in parasitemic cattle compared to aparasitemic cattle. This may be because of the lower PCV resulted from the devastating condition of the trypanosome [37]. Reduced diet and intercurrent digestive tract parasite disease may be also predisposing to the total low PCV. Therefore, in the absence of these two aspects, anemia is the best pointer of trypanosomosis.

## 5. Conclusions and Recommendations

Generally, this study indicates that the proportion of bovine trypanosomosis in Hawa Galan woreda was relatively high and represents a risk to cattle owners in this area because of reduction in yield and productivity of cattle. From the results of this study, the predominant trypanosome species in animals of this study were *T. congolense* and *T. vivax*. While further research is required to support the results of the current study, the following points are suggested to improve the management of trypanosomosis: vector challenge in the area could be minimized via wide application of traps and insecticide-impregnated targets or by use of available chemicals on the cattle; regular screening of cattle for trypanosomosis and early treating of positive cattle with trypanocides are essential; teaching cattle owners, particularly those located in the highest vector burden areas, is important to minimize the risk of interaction of cattle with vectors; and the region could also be equipped with sufficient veterinary service, especially Lemlem PA.

## Figures and Tables

**Table 1:** Proportion of trypanosomosis-positive cattle based on the Buffy coat test result in relation to origin.

Origin	Cattle examined	Positive result	Proportion (%)	*χ* ^2^	*P* value
Lemlem	120	15	12.5		
Mada walkituma	100	3	3	12.15	0.002
Ifa jiru	208	8	3.8		
**Total**	**428**	**26**	**6**		

Bold indicates *P* value = the probability value (significance level) and indicates strength of associations among dependent and independent variables. Since *P* value is 0.002 which is <0.05, the relationship based on origin is significant.

**Table 2:** Proportion of bovine trypanosomosis on the basis of the body condition of the animal.

Body condition	Cattle examined	Positive result	Proportion (%)	*χ* ^2^	*P* value
Poor	134	16	11.9		
Medium	130	7	5.38	13.3692	0.001
Good	164	3	1.8		
**Total**	**428**	**26**	**6**		

Bold indicates *P* value = the probability value (significance level) and indicates strength of associations among dependent and independent variables. Since *P* value is 0.001 which is <0.05, the relationship based on body condition is significant.

**Table 3:** Proportion of bovine trypanosomosis based on of the sex of animals.

Sex	Cattle tested	Positive result	Proportion (%)	*χ* ^2^	*P* value
Male	209	14	6.7		
Female	219	12	5.48	0.2786	0.598
**Total**	**428**	**26**	**6**		

Bold indicates *P* value = the probability value (significance level) and indicates strength of associations among dependent and independent variables. Since *P* value is 0.598 which is >0.05, the relationship based on sex is not significant.

**Table 4:** Proportion in relation to different age groups.

Age category	Cattle examined	Positive result	Proportion (%)	*χ* ^2^	*P* value
<2 years	84	2	2.38	3.1261	0.210
2–5 years	154	9	5.85		
>5 years	190	15	7.9		
**Total**	**428**	**26**	**6**		

Bold indicates *P* value = the probability value (significance level) and indicates strength of associations among dependent and independent variables. Since *P* value is 0.210 which is >0.05, the relationship based on age is not significant.

**Table 5:** Proportion of PCV of parasitemic and aparasitemic cattle in the study area.

Status	Cattle examined	PCV < 24%	PCV ≥ 24%	*χ* ^2^	*P* value
Parasitemic	26	23 (10.45%)	3 (1.44%)	15.224	≤0.001
Aparasitemic	402	197 (89.54%)	205 (51%)		
**Total**	**428**	**220 (51.4%)**	**208 (48.6%)**		

Bold indicates *P* value = the probability value (significance level) and indicates strength of associations among dependent and independent variables. Since *P* value is 0.001 which is <0.05, the relationship based on status of PCV is significant.

**Table 6:** Effect of the different risk factors associated with trypanosomosis using multivariate logistic regression.

Variables	No. sampled	Positive result	Proportion (%)	Odds ratio	*P* value	95% CI
*Sex*						
Male	209	14	6.7	1.34	0.47	0.6–3
Female	219	12	5.48	Ref^∗^	―	―
Total	428	26	6			
*Age category*						
≤2	84	2	2.38	Ref^∗^	―	―
2–5	154	9	5.85	2.67	0.22	0.55–12.8
≥5	190	15	7.9	2.55	0.23	0.56–11.6
Total	428	26	6			
*Body condition*						
Poor	134	16	11.9	Ref^∗^	―	―
Medium	130	7	5.38	0.4	0.062	0.16–1
Good	164	3	1.8	0.14	0.003	0.04–0.5
Total	428	26	6			

*P* value = the probability value (significance level) and indicates strength of associations among dependent and independent variables. CI = confidence interval; Ref = reference.

## Data Availability

The data used to support the findings of this study are included in this published article and will be available from the corresponding author upon request.

## References

[1] World Organisation for Animal Health (2008). *Epizooties (OIE), Trypanosomosis. Terrestrial Manual. Office International des. Epizooties (OIE)*.

[2] Food and Agricultural Organization (FAO) (1982). *Office International des. Epizooties (OIE)*.

[3] Food and Agricultural Organization (FAO) (2002). *Food, Agriculture and Food Security: The Global Dimension*.

[4] Bitew M., Amedie Y., Abebe A., Tolosa T. (2011). Proportion of bovine trypanosomosis in selected areas of JabiTehenan district, West Gojam of Amhara regional state, Northwestern Ethiopia. *African Journal of Agricultural Research*.

[5] Food and Agricultural Organization (2002). *Food, Agriculture and Food Security, the Global Dimension*.

[6] Ministry of agriculture and rural development of the government of Ethiopia (MOARD), tsetse and trypanosomosis prevention and control strategies.

[7] Abebe G. (2005). Trypanosomes in Ethiopia. *Ethiopian Journal of Biological Sciences*.

[8] Mottelib A. A., Hosin H. I., Mould I. (2005). *El-sherif, A.M., Abo-Zeid, Comparative Evaluation of Various Diagnostic Techniques for T*.

[9] Holand W., Deeris G. M., Verckuysse J. (2004). Prevalace of *T. evansi* in water buffalos inremote areas in northern Vietnam using PCR and seriologically methods. *Trop. Animals Health Prod.*.

[10] Schofield C. J., Maudlin I. (2001). Trypanosomosis control. *International Journal for Parasitology*.

[11] Donelson J. E. (2003). Antigenic variation and the African trypanosome genome. *Acta Tropica*.

[12] Akol G. W. O., Authie E., Pinder M., Moloo S. K., Roelants G. E., Murray M. (1986). Susceptibility and immune responses of Zebu and taurine cattle of West Africa to infection with transmitted by. *Veterinary Immunology and Immunopathology*.

[13] Shimelis D., Arun K. S., Getachew A. (2008). Assessment of trypanocidal drug resistance in cattle of the abay (blue nile) basin areas of northwest Ethiopia. *Ethiopian Veterinary Journal*.

[14] Dagnachew S., Terefe G., Abebe G., Barry D. J., McCulloch R., Goddeeris B. M. (2014). *In vivo* experimental drug resistance study on *Trypanosoma vivax* isolates from tsetse infested and non-tsetse infested areas of Northwest Ethiopia. *Acta Tropica*.

[15] Degneh E., Ashenafi H., Kassa T. (2019). Trypanocidal drug resistance: a threat to animal health and production in Gidami District of Kellem Wollega zone, Oromia regional state, Western Ethiopia. *Preventive Veterinary Medicine*.

[16] Dayo G.-K., Bengaly Z., Messad S. (2010). Prevalence and incidence of bovine trypanosomosis in an agro-pastoral area of southwestern Burkina Faso. *Research in Veterinary Science*.

[17] Duguma R., Tasew S., Olani A. (2015). Spatial distribution of glossina sp. and trypanosoma sp. in south-western Ethiopia. *Parasites & Vectors*.

[18] Degneh E., Shibeshi W., Terefe G., Aseres K., Ashenafi H. (2017). Bovine trypanosomosis: changes in parasitemia and packed cell volume in dry and wet seasons at Gidami District, Oromia regional state, Ethiopia. *Acta Veterinaria Scandinavica*.

[19] Lelisa K., Shimeles S., Bekele J., Sheferaw D. (2014). Bovine trypanosomosis and its fly vectors in three selected settlement areas of Hawa-Gelan district, western Ethiopia. *The Onderstepoort Journal of Veterinary Research*.

[20] Lelisa K., Damena D., Kedir M., Feyera T. (2015). Prevalence of bovine trypanosomosis and apparent density of tsetse and other biting flies in Mandura District, Northwest Ethiopia. *Journal of Veterinary Science and Technology*.

[21] Galan Woreda Agricultural Office. (HGWAO) (2015). *Hawa Galan Woreda Agricultural Office Annual Report*.

[22] Nicholson M. J., Butterworth M. H. (1986). *A Guide to Scoring of Zebu Cattle*.

[23] Thrusfield M. (2005). *Veterinary Epidemiology*.

[24] Office International des (2008). *Epizooties (OIE), “Standardized Techniques for the Diagnosis of Tsetse Transmitted Trypanosomosis*.

[25] Leak S. G. (1999). *Tsetse Biology and Ecology: Their Role in the Epidemiology and Control of Trypanosomosis*.

[26] Paris J., Murray M., Mcodimba F. (1982). A comparative evaluation of the parasitological technique currently available for the diagnosis of African Trypanosomosis in Cattle. *Acta Tropica*.

[27] Efrem D., Bashatu F., Bacha B., Addisalem H., Misgana D. (2013). Proportion of bovine trypanosomosis in LaloKile district KelemWollegaZone,Oromia regional state, western Ethiopia. *Acta Parasitological Globalis*.

[28] Alemayehu B., Bogale B., Fentahun T., Chanie M. (2012). Bovine Trypanosomosis: a threat to cattle production in chena district, Southwest Ethiopia. *Open Journal of Animal Sciences*.

[29] Dano T., Benti D., Mukarim A. (2014). Proportion of bovine trypanasomosis in GutoGida district of EastWollega zone, Oromia regional state, Ethiopia. *Global Journals Incorporated*.

[30] Tadele K., Siyum G., Zelalem A., Benti D. (2014). Epidemiological survey of bovine trypanosomosis in SayoDistrict of KellemWollega zone, western Ethiopia. *American-Eurasian Journal of Scientific Research*.

[31] Abraham Z., Tesfaheywet Z. (2012). Proportion of bovine trypanosomosis in selected district of Arba minch, SNNP, southern Ethiopia. *Global Veterinary Services & Agriculture*.

[32] Fedesa H., Assefa K. .., Tekalegn D. (2015). Study on spatial distribution of tsetse fly and proportion of bovine trypanosomosis and other risk factors: case study in darimu district, ilu aba bora zone, western Ethiopia. *The Journal of Pharmacy and Alternative Medicine*.

[33] Tafese W., Melaku A., Fentahun T. (2012). Proportion of bovine trypanosomosis and its vectors in two districts of East Wollega Zone, Ethiopia. *The Journal of Pharmacy and Alternative Medicine*.

[34] Murray M. Trypanotolerance, its criteria and genetic and environmental influence.

[35] Torr S. J., Mangwiro T. N. C., Hall D. R. (2006). The effects of host physiology on the attraction of tsetse (Diptera: Glossinidae) and Stomoxys (Diptera: muscidae) to cattle. *Bulletin of Entomological Research*.

[36] Teka W., Terefe D., Wondimu A. (2012). Proportion study of bovine trypanosomosis and tsetse density in selected villages of Arbaminch, Ethiopia. *Journal of Veterinary Medicine and Animal Health*.

[37] Radostits O. M., Gay C. C., Hinchcliff K. W., Constable P. D. (2007). *Veterinary Medicine, A Textbook of the Disease of Cattle, Sheep, Goat, Pigs and Horses*.

